# Rainbow of Chaos: A study into the Theory and Practice of Integrated Primary Care: Pim P. Valentijn, [S.l.: s.n.], 2015 (Print Service Ede), pp. 195, Doctoral Thesis Tilburg University, The Netherlands, ISBN: 978-94-91602-40-5

**DOI:** 10.5334/ijic.2465

**Published:** 2016-05-03

**Authors:** Pim P. Valentijn

**Affiliations:** Harderwijk, The Netherlands

**Keywords:** Integrated care, Primary care, Care coordination, Organization models, Continuity of care, Multidisciplinary care, Collaboration

## Abstract

This thesis aimed to contribute to a better understanding of what integrated primary care is, and how it can be achieved by focussing on the collaboration processes that underlie the development of integrated primary care. The first part of this thesis operationalized the concept of integrated care from a primary care perspective. The second part of this thesis described the collaboration mechanisms among integrated care projects that were part of a national integrated primary care study in The Netherlands.

## Introduction

Integrated primary care services are considered a vital strategy for maintaining sustainable and affordable healthcare provisions. However, a solid scholarly explanation about the concept of integrated primary care and knowledge of its accompanying collaboration processes for success is limited. This thesis aimed to contribute to a better understanding of what integrated primary care is, and how it can be achieved by focussing on the collaboration processes that underlie the development of integrated primary care. This thesis was divided into two parts to address the leading research questions:

1. What is integrated care in the context of primary care?
2. What is the role of collaboration in the development of integrated primary care?

## Main findings

### Part I: What is integrated care in the context of primary care?

The first part of the thesis concerned the reconfiguration and operationalization of the concept of integrated care from a primary care perspective. Based on the theoretical assumptions of integrated care and primary care, the Rainbow Model of Integrated Care (RMIC) was developed to grasp the complex and multi-dimensional nature of integrated care. This model distinguishes two primary care guiding principles, namely person-focused and population-based, and six domains of integrated care, namely clinical, professional, organisational, system, functional and normative integration. In the RMIC, integrated care plays an interconnected role at the micro level of clinical integration, the meso level of professional and organisational integration, and the macro level of system integration. Functional and normative integration are conceptualized as enablers for linking the various levels. The RMIC visualises that integrated care can be pursued at different levels within a system to facilitate the continuous, comprehensive, and coordinated delivery of services for individuals and populations.

Further insight into the underlying features of the various domains was needed in order to make the model applicable for evaluation purposes. Firstly, a literature review and thematic analysis procedure were conducted to refine the RMIC into a taxonomy of fifty-nine key features. Thereafter, Delphi studies among experts from various countries were performed to develop an international consensus-based taxonomy. The results showed that the experts were particularly focused on the features that are part of the clinical, professional and organisational domains of integration, while features that are part of the ‘macro’ system integration domain were generally neglected. With the results of the Delphi studies the taxonomy was revised resulting in twenty-one key features organised into three main categories: scope (person-focused vs. population-based), type (clinical, professional, organisational and system) and enabler (functional vs. normative). The three main categories of the taxonomy provide a crucial differentiation for the clarification and interpretation of practical examples of integrated care. For example, specifying the scope of an integrated care initiative as either person-oriented or population-oriented can help understanding and describing the guiding principles and objectives of the initiative. In addition, the types of integration process can be used to explore the (dis)similarity of integration mind-sets between stakeholders at the clinical, professional, organisational and system levels of integration. Finally, the enablers can assist in clarifying and interpreting the technical (functional) and cultural (normative) conditions needed to achieve common goals and reach optimal results with an integration initiative. Profiling integrated care initiatives along the taxonomy makes it possible to to obtain comprehensive and systematic information, and promotes the learning and sharing of best practices.

### Part II: What is the role of collaboration in the development of integrated primary care?

The second part of the thesis described the collaboration mechanisms among integrated care projects that were part of a national integrated primary care study in The Netherlands. The longitudinal relationship between collaboration processes and the perceived effectiveness at the strategic level was studied in a sample of 59 integrated care projects. Several collaboration processes played a role at the start and during the development of the projects, namely mutual gains, relationship dynamics, organisational dynamics (shared control) and process management. Both mutual gains (the acknowledgement of various interests between partners) and process management (the steering of the collaboration process) were prerequisites for the successful development of integrated care projects. Over time an increase in relationship capital among actors was associated with successful development of integrated care. These results suggested that trust-based collaboration processes (i.e. mutual gains and relational capital) are more important than control-based processes (i.e. process management) for the development of integrated care initiatives.

A subsample of 42 integrated primary care projects was used to assess how changes in collaboration processes over time relate to the degree of integration effectiveness as perceived by stakeholders at the professional, organisational and system levels. The (dis)similarity of integration mind-sets between stakeholders was used to create a typology of integrated primary care projects. Analyses resulted in a ‘United Integration Perspectives (UIP)’ subgroup, a ‘Disunited Integration Perspectives (DIP)’ subgroup, and a ‘Professional-oriented Integration Perspectives (PIP)’ subgroup. Changes in collaboration processes in the subgroups were contrasted over time as well as the final perceived effectiveness rates among stakeholders. The projects characterised by an UIP increased the most in both trust-based (mutual gains and relationship dynamics) and control-based (organisational dynamics and process management) collaboration processes and had the highest overall effectiveness rates among stakeholders. In contrast, projects characterised by a DIP decreased on collaboration processes and had the lowest overall effectiveness rates among stakeholders. Projects characterised by a PIP increased in control-based collaboration processes (organisational dynamics and process management) and achieved the highest effectiveness rates among professionals. These findings highlighted the need to have similar integration mind-sets among the stakeholders for an integrated care project to be effective. To conclude, both trust-based and control-based collaboration processes are required to align disunited integration mind-sets. During the development of an initiative, collaboration processes are essential for achieving the collaborative advantage of an integrated care initiative.

## Discussion

**Figure 1:**
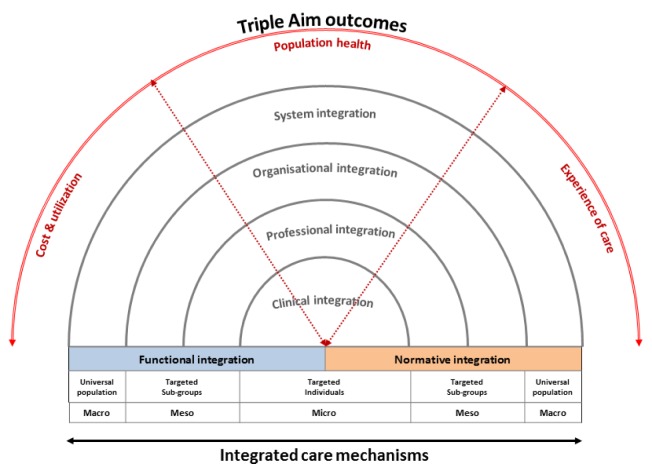
The revised RMIC, value-based integrated care. Source: Valentijn (p. 160, 2015).

The first part of the thesis provides a theory underpinning how clinical, professional, organisational and system integration efforts act at several levels (micro, meso and macro) and can be defined from multiple stakeholder perspectives (patients, professionals, managers and policymakers). The theoretical analysis also led to the understanding that both ‘hard’ functional (e.g. IT, financial incentives) and ‘soft’ normative (e.g. cultural values) mechanisms are essential for enabling the implementation of integrated care. The second part of the thesis showed that normative collaboration mechanisms at the strategic level influence the development of integrated care. The theoretical and empirical findings of the thesis contributed to the unravelling of the concept of integrated care. However, further work is needed to understand how integrated care mechanisms act as a means for improved health and cost-related outcomes. The link between the theoretical rationale of the RMIC and the three interdependent outcomes of the Triple Aim framework must be examined in order to bridge this gap and identify the three-dimensional value perspective (patient, social and economic) of integrated care, see Figure 1.

## Recommendations for research and practice

This thesis provides valuable knowledge for the implementation of integration strategies in a primary care context. The RMIC and related taxonomy can be applied by policymakers, commissioners, managers, professionals and patient organisations to guide the planning and the design of integrated care initiatives. The main implication of the thesis is that developing integrated care is a complex change strategy which typically involves multiple integration efforts at multiple levels. This implies that, in line with a complexity philosophy of science, more emphasis needs to be placed on theorising, studying and modelling interaction patterns within and between the clinical, professional, organisational and system levels of integration. Methods, such as social network analyses can be a useful aid to study these complex interaction patterns further.

## Conclusion

The development of integrated primary care is a complex process involving activities at multiple levels that are not predictable nor linear, but also not chaotic. Without doubt, no magic blueprint exists for the successful organisation of integrated care best suited for all contexts, settings and circumstances. Instead, integrated care is more of an ‘art form’ founded on a colourful pallet of values and perceptions arising from several political, organisational, professional and clinical fields. Although the thesis demonstrates that constructive relationships are fundamental to developing integrated care in practice, it is naive to assume that only ‘trust’ can bind the system together. Relying on normative relational approaches without the proper supporting functional tools and incentives does not seem to be a sustainable solution for the long-term development of integrated care. Grounding integrated primary care will require multiple perspectives that unite in a person-focused as well as population-based and value-driven vision.

The results presented in this review are based on the author’s thesis presented at Tilburg University, The Netherlands on 16 December 2015.

Full text available from: URL: https://pure.uvt.nl/portal/files/9272073/Valentijn_Rainbow_16_12_2015.pdf.

## Competing Interests

The author declares that they have no competing interests.

## Author Information

Dr. Pim Valentijn is a principal investigator and consultant in the field of value-based integrated care. Valentijn’s research focus is on assessing the value of integrated services models in primary and secondary care. His specific skills is to translate scientific knowledge into practical tools for care providers, purchasers of care, policymakers and patients. In 2014, the International Foundation for Integrated Care (IFIC) awarded Valentijn its Integrated Care Award, acknowledging his publication about the Rainbow Model of Integrated Care for its outstanding contribution to the science and practice of integrated care. He is an advisor to various health care organizations, health care industries and policy boards. Valentijn holds a Ph.D. from Tilburg University and a M.Sc. from VU University.
